# Ferroptosis Involvement in Glioblastoma Treatment

**DOI:** 10.3390/medicina58020319

**Published:** 2022-02-20

**Authors:** Andrei-Otto Mitre, Alexandru Ioan Florian, Andrei Buruiana, Armand Boer, Ioana Moldovan, Olga Soritau, Stefan Ioan Florian, Sergiu Susman

**Affiliations:** 1 Department of Morphological Sciences, Iuliu Hațieganu University of Medicine and Pharmacy, 8 Victor Babes Street, 400012 Cluj-Napoca, Romania; andrei.mitre97@gmail.com (A.-O.M.); armandboer.jr@gmail.com (A.B.); ioana.orlea@gmail.com (I.M.); sergiu.susman@umfcluj.ro (S.S.); 2 Department of Neurosurgery, Iuliu Hațieganu University of Medicine and Pharmacy, 8 Victor Babes Street, 400012 Cluj-Napoca, Romania; stefanfloriannch@gmail.com; 3 Department, of Neurosurgery, Emergency County Hospital, 3-5 Clinicilor Street, 400006 Cluj-Napoca, Romania; 4 Department of Medical Oncology, Prof. Dr. I. Chiricuta Oncology Institute, 400015 Cluj-Napoca, Romania; andrei.buruiana@yahoo.com; 5 Research Department, Prof. Dr. I. Chiricuta Oncology Institute, 400015 Cluj-Napoca, Romania; soritau@iocn.ro; 6 Department of Pathology, IMOGEN Research Center, Louis Pasteur Street, 400349 Cluj-Napoca, Romania

**Keywords:** ferroptosis, glioblastoma, lipid peroxidation, cell death

## Abstract

Glioblastoma multiforme (GBM) is one of the deadliest brain tumors. Current standard therapy includes tumor resection surgery followed by radiotherapy and chemotherapy. Due to the tumors invasive nature, recurrences are almost a certainty, giving the patients after diagnosis only a 12–15 months average survival time. Therefore, there is a dire need of finding new therapies that could potentially improve patient outcomes. Ferroptosis is a newly described form of cell death with several implications in cancer, among which GBM. Agents that target different molecules involved in ferroptosis and that stimulate this process have been described as potentially adjuvant anti-cancer treatment options. In GBM, ferroptosis stimulation inhibits tumor growth, improves patient survival, and increases the efficacy of radiation and chemotherapy. This review provides an overview of the current knowledge regarding ferroptosis modulation in GBM.

## 1. Introduction

Glioblastoma multiforme (GBM) is a grade IV glioma, the most common malignant primary brain tumours in adults [1,2]. Its primarily characteristic is its high malignancy and invasion ability that renders a complete surgical resection impossible [3]. Therefore, current treatment options involve maximal surgical resection of the tumour, followed by chemotherapy and radiotherapy that are aimed at destroying the remaining cells [4,5]. However, these remaining cells can activate survival pathways and acquire resistance to both chemo and radiation therapy, most patients having tumour recurrence, that are often times more aggressive than the initial tumour [6,7,8]. This is why, even with this full treatment scheme, GBM patients have an average survival time between 12- and 15-months following diagnosis [9,10]. Current research is focusing on reducing the tumoral cells survival, either by targeting specific pathways used by the tumour for survival or by targeting pathways that offer the tumour resistance to chemo and radiation therapy [11,12].

Ferroptosis is a newly described form of regulated cell death [13]. Compared with apoptosis, necroptosis and pyroptosis, ferroptosis is iron dependent, triggered by intra-cellular alterations in iron metabolism that leads to lipid peroxidation and subsequently cellular death [14]. This mechanism has shown to be involved in a large number of pathologies, including inflammation, ischemic brain injuries and cancers, among which GBM is one [15,16,17,18,19].

This review aims to summarize the current knowledge regarding the ferroptosis process in GBM and the influence of ferroptosis pathways modulation on cancer cell activity.

## 2. Materials and Methods

The purpose of this review is to offer an overview of the current knowledge regarding the ferroptosis process in glioblastoma. We searched electronic databases of Medline (PubMed, PubMed Central) by using the terms ‘glioblastoma’ or ‘GBM’ and ‘ferroptosis’ or the molecules involved in the ferroptosis pathway: ‘iron’, ‘transferrin’, ‘transferrin receptor’, ‘TfR’, ‘STEAP’, ‘divalent metal transporter’, ‘DMT’, ‘PCBP’, ‘ferritin’, ‘ferroportin’, ‘system xc’, ‘SLC3A2’, ‘SLC7A11’, ‘cysteine’, ‘GSH’, ‘GPX4’, ‘glutathione’, ‘lipid peroxidation’, ‘ACSL4’, ‘ALOXE3’. Afterward, using a cross-reference search, we evaluated further articles linking ferroptosis to cancer progression and treatment. We have considered both clinical and experimental studies (both in vivo and in vitro).

## 3. The Ferroptosis Pathway

**Figure 1:**
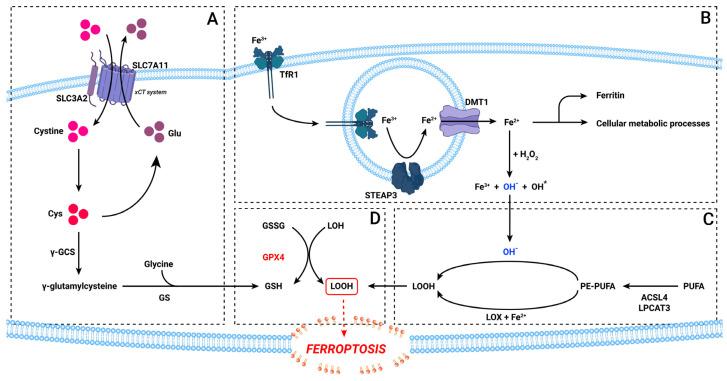
The ferroptosis pathway. (**A**): the xCT-cysteine pathway; (**B**): the iron pathway; (**C**): the PUFA and lipid peroxidation pathway; (**D**): the GPX4 involvement. Abbreviations: ACSL4, long-chain-fatty-acid-CoA ligase 4; Cys, cysteine; DMT1, divalent metal transporter 1; Fe, iron; γ-GCS, γ-glutamylcysteine synthetase; γ-glutamylcys, γ-glutamylcysteine; Glu, glutamate; GPX4, glutathione peroxidase 4; GS, glutathione synthetase; GSH, glutathione; GSSG, glutathione disulphide; LOH, lipid alcohol; LOOH, lipid peroxide; LOX, lipoxygenase; LPCAT3, lysophosphatidylcholine acyltransferase 3; PE, phosphatidylethanolamine; PUFA, polyunsaturated fatty acids; SLC3A2, solute carrier family 3 member 2; SLC7A11, solute carrier family 7 member 11; STEAP3, six-transmembrane epithelial antigen of the prostate 3; TFR1, transferrin receptor 1; xCT, cystine/glutamate antiporter.

Cell death is a vital component of the normal animal cell development and homeostasis, it’s absence or dysregulation being involved in a series of immunological, developmental and neurological diseases, including excessive proliferative diseases such as cancers [20]. Although cysteine depletion forms of cells deaths have been observed for some time, ferroptosis was only recently described as a form of non-apoptotic cell death [13,21]. Ferroptosis is regulated by a series of very complex processes [14]. For this review, we will mainly focus on the core mechanism leading to ferroptosis: iron-related reactive oxygen species production, cysteine depletion and lipid peroxidation (Figure 1).

Iron in the cellular metabolism is presented in 2 forms: the ferrous cation (Fe^2+^) and ferric cation (Fe^3+^). Fe^3+^ is bound in the blood to plasma transferrin that carries it to the cells. Here, it attaches to the transferrin receptor (TfR) 1, the complex being endocytosed into the cell. Fe^3+^ is reduced to Fe^2+^ by 6-transmembrane epithelial antigen of the prostate 3 (STEAP3), which then enters the cytoplasm via divalent metal transporter 1 (DMT1) of the endosomal membrane [22,23]. Cytoplasmatic free Fe^2+^ is metabolic active and can be involved in a series of enzymatic processes, including DNA synthesis, cell cycle progression and angiogenesis, sequestered into ferritin by poly r(C)-binding protein (PCBP) 1 and 2 or taking place in the Fenton reaction [24]. Ferrous iron leaves the cells via ferroportin, which acts as a negative regulator of ferroptosis [25]. During the Fenton reaction, Fe^2+^ binds to hydrogen peroxide, which results in the generation of hydroxide and a hydroxyl radical that is a reactive oxygen species (ROS) [26]. ROS production leads to lipid peroxidation and loss of membrane permeability (Figure 1B) [27].

Another cnetral mechanism that leads to lipid peroxidation is via the cysteine/GSH depletion pathway. L-cystine enters the cells via the x_c_^−^ exchanger with glutamate, which is composed of SLC7A11 and SLC3A2 carriers [28]. From here, it can be involved in a series of metabolic transformations, including the production of reduced glutathione (GSH). This happens by transforming L-cystine in γ-glutamylcysteine with the help of γ-glutamylcysteine synthase and then transforming it into GSH by glutathione synthase and glycine [29]. GSH is important in maintaining the cellular redox balance, as it is an ROS scavenger, being oxidized to glutathione disulphide (GSSG) (Figure 1A,D) [30].

Polyunsaturated fatty acids (PUFAs) are key element in ferroptosis. For PUFAs to lead to ferroptosis, first they must be esterified. Not all PUFAs act as substrate for the ferroptosis-induced lipid oxygenation, but mainly phosphatidylethanolamine (PE) PUFAs [31]. These PE-PUFAs are formed by acyl-CoA synthetase long-chain fatty member 4 (ACSL4) and lysophospatidylcholine acyltrasferase 3 [32]. By the action of 15-lipoxygenases (LOX) and Fe^2+^, PE-PUFAs are oxidized into lipid peroxides (Figure 1C) [33].

ROS accumulation via disruption of these pathways leads to the formation of lipid peroxides. Lipid peroxides are normally transformed in lipid alcohols by glutathione peroxidase 4 (GPX4) that uses 2 GSH molecules as substrate [34]. This step represents the main protective mechanism against ferroptosis (Figure 1D).

Besides GPX4 there are other cellular protective mechanisms against ferroptosis. In GPX4-knockdown cells, a protective mechanism was represented by the activation of ferroptosis suppressor protein 1 (FSP1), also known as apoptosis-inducing factor mitochondria-associated 2 (AIFM2) or p53-responsive gene (PRG3) [35]. FSP1 is a NADPH-dependent coenzyme Q_10_ (CoQ_10_) oxidoreductase that acts by converting ubiquinone (CoQ_10_) from the cellular wall into ubiquinol. Ubiquinol then acts as a ROS scavenger and can prevent ferroptosis-characteristic lipid peroxidation [36]. Further research is needed to establish the exact mechanism of FSP1 anti-ferroptosis mechanism, as 1 study showed that FSP1 can block drug-induced ferroptosis via an CoQ_10_ independent pathway [37]. Shi et al. showed that FSP1 levels were significantly associated with a lower overall survival and disease-free interval in several cancer types and could represent prognostic markers [38].

It is important to find and understand these alternate protective pathways because strategies aimed at promoting ferroptosis in cancer cells via up-regulating GPX4 could potentially be rendered ineffective by the compensatory activation of these other mechanisms.

Any modification of the levels and regulation of these pathways can hinder or promote ferroptosis. Increased intracellular Fe^2+^ and a decrease in GSH production increase the amount of ROS and lipid peroxidation; GPX4 inhibition disrupts the transformation of lipid peroxides into lipid alcohol and an increased level of lipid peroxides damages the structure of organelles and cell membranes, causing a loss of permeability and leading to cell death via ferroptosis.

## 4. Ferroptosis in Cancer Treatment

Current anti-cancer therapies aim to reduce or completely eradicate the tumour cells. This is conventionally conducted by surgical removal of the tumour, followed by radiation therapy and chemotherapy, as well as other forms of targeted therapies (Shah, Viral, 2018). The common denominator of these strategies is generally the aim of targeting a gene or protein that can trigger the apoptosis process. This involves the permeabilization of the mitochondrial outer membrane, which triggers the release of cytochrome c, the formation of the apoptosome the activation of caspase-9, concluding caspase-3 activation [39]. Caspase-3 triggers the execution pathway that involves poly (ADP-ribose) polymerases cleavage with DNA degradation, cytoskeletal reorganization, nuclear fragmentation and ultimately, the formation of apoptotic bodies and cell death [40,41,42]. Therefore, tumour development inhibition by caspase-3 activation is 1 of the main therapeutic targets of current anti-cancer therapies [43,44].

However, one of the main concerns regarding this strategy is represented by the cancer cells developing resistance to apoptosis [45]. The most common anti-apoptotic mechanisms developed by cancer cells are the expression of BCR-ABL or the silencing of tumour suppressor p53 [46,47,48]. In this regard ferroptosis presents as a valuable alternative, as it is a non-apoptotic form of cell death and could aid current anti-cancer treatments [49].

Cancer cells have a higher iron metabolic demand, making them more susceptible than normal cells to ferroptosis [49]. Recent studies have highlighted the involvement of ferroptosis in several cancer types, including breast cancer, pancreatic cancer, hepatocellular carcinoma, renal cell carcinoma and glioblastoma [50,51,52,53,54]. Ferroptosis sensitivity of cancer cells could be related to Ras-mitogen-activated protein kinase (MEK) activation. This contributes to the up-regulation of transferrin receptor 1 and the increase in intra cellular iron levels, and also to the additional formation of ROS by cystine inhibition [55,56]. There are many other molecules that are discovered to be involved in ferroptosis in different cancer cells, many of which could pose as a therapeutic target for future studies, including nuclear factor erythroid 2-related factor 2, heat shock-factor-1 and Beclin 1 [18]. Several studies have shown that in glioblastoma cell lines increasing ferroptosis activity resulted in decreased tumour growth and aggressiveness [57,58,59,60,61,62]. This could pose as a potential therapeutic strategy in GBM, aiding the current post-operative treatments [63,64,65,66].

However, it is important to note that most of these results are in vitro. Ferroptosis activation, by destroying the cellular membranes, determines the outburst of intra-cellular elements and ROS, which trigger a local inflammatory response that could damage the surrounding healthy tissue [67]. In non-alcoholic fatty liver disease and in ischemia-reperfusion injury ferroptosis played a pro-inflammatory role, aiding disease progression. In these cases, ferroptosis inhibition resulted in a better outcome [68,69]. Moreover, ferroptosis contributes to cyclooxygenase 2 activation that leads to the formation of prostaglandins and inflammation [70]. In the brain, several neurodegenerative diseases were linked to ferroptosis, including multiple sclerosis, Alzheimer’s disease, Parkinson’s disease and Huntington’s disease [71]. In all of these pathologies, ferroptosis was involved in a part of the neuroinflammatory process. These results should caution future research into evaluating the possible neurological damage possibly associated with ferroptosis modulation in GBM subjects.

In the following section, we will describe the influence of modulating the ferroptosis pathway in GBM.

## 5. Ferroptosis in Glioblastoma

### 5.1. Iron Metabolism

GBM cells appear to have high free iron levels, even higher than GBM cancer stem-cells, which are probably able to store more as ferritin [72]. So far, the exact mechanisms and relationship that iron metabolism dysregulation has to cancer progression is unclear, but amending it could represent a promising strategy in improving the outcome of anti-cancer treatments [73]. Iron-related gene expression (hepcidin, TfR1 and TfR2) is different between normal human brain tissue and brain tumours, either down- or up-regulated [74,75]. In GBM sample tissues, TfR levels were higher compared to meningiomas and other brain tumours, with the exception of one tissue sample from a patient that underwent radiation therapy [76]. TfR2 is highly expressed in glioblastoma cell lines, which contributes to cell proliferation. Additionally, high TfR2 levels are associated with a better sensitivity for temozolomide and thus a more favourable prognosis [77].

STEAP3 is involved in iron homeostasis by reducing Fe^3+^ to Fe^2+^ that can be used by the cell [78]. However, the STEAP3 protein is also involved in other processes, including the inflammatory response, being vital for the Toll-Like Receptor 4-mediated macrophage production of chemoattractant protein-5, interferon-beta and interferon induced protein-10 [79,80]. In GBM cells, STEAP3 is highly expressed compared with normal brain tissues. This makes STEAP3 expression as a potential prognosis marker, poorer overall survival being associated with an increased STEAP3 level [81,82]. Furthermore, STEAP3 promotes TfR expression and induces mesenchymal transition. Its expression was directly correlated with increased cell proliferation, invasion, and sphere formation in vitro and with increased tumour growth in vivo [83].

DMT1 is associated with increased intracellular iron levels and iron accumulation in the brain and is currently studied as a molecule involved in neurodegenerative diseases [84]. In an experimental C6 glioma cells rat model, propofol administration reduced DMT1 expression in the glioma cells. The reduction was further associated with a significant decrease in GSH/GSSG ratio and of ROS. Additionally, tumour cells in the propofol groups had a lower tumoral cell count [85]. These results could link the expression of DMT1 and iron levels to ROS production and tumour proliferation, presenting DMT1 as a potential therapeutical target. During temozolomide (TMZ) treatment of GBM, DMT1 levels were elevated, associated with an increase in ROS via the ferroptosis pathway. Therefore, TMZ can suppress cell growth via the ferroptosis pathway by targeting DMT1 expression [86].

PCBP2 is an important element in iron homeostasis, as well as in posttranscriptional and translational regulation. It is upregulated in glioma tissues and cell lines and its knockdown inhibits glioma growth [87]. PCBP2 inhibitor microRNA-214 reduced glioma growth and proliferation [88].

In cancer patients, ferritin is detected in higher levels in the serum, correlating with a poorer prognosis and outcome. This illustrates the importance of iron and iron metabolism in cancer progression and possible resistance to therapy [89]. In GBM patients, ferritin levels in the serum and cerebrospinal fluid were higher than in patients without tumours. This excess ferritin appeared to be produced by the GBM cells [90].

### 5.2. The xCT System

Cysteine deprivation is an important inducer of ferroptosis and greatly contributes to the ferroptosis in GBM [91]. The cystine/glutamate xc- system antiporter is a heterodimer composed of the subunit SLC3A2, with the role of anchoring and stabilizing the SLC7A11 subunit, and SLC7A11, which is mediating the antiporting activity. Throughout the literature, this system is also referred to as simply SLC7A11 or xCT. For this reason, we will refer from now on to the antiporter as xCT. As mentioned before, this system is important in ferroptosis because its ability to regulate intracellular cystine intake and subsequently cysteine availability [92]. In GBM, xCT plays an important role in tumour survival and progression [93]. In the study of Takeuchi et al., including 40 GBM patients, xCT expression was correlated with the clinical outcome. Stronger xCT expression was significantly associated with a shorter progression-free survival and a shorter overall survival [94].

The tumour suppressor and transcription factor p53 is usually deregulated in GBM, increasing the tumours invasiveness, angiogenesis and regulating cellular metabolism. As this factor is downregulated, p53 reactivators could pose as a possible therapeutic strategy [95]. One study showed that there is a negative correlation between the expression of p53 and SLC7A11 in GBM. p53 can directly suppress *SLC7A11* gene expression. Treatment with p53 reactivator decreased xCT activity and tumour growth; therefore, these treatments were able to influence both the p53 and the xCT systems [96].

In glucose deprivation environments, high cell density and epidermal growth factor treatment upregulated xCT in GBM cell lines, causing tumour death [97,98]. The effect is explained by an increase in cysteine levels, NADPH depletion and subsequent ROS accumulation [99,100]. However, these in vitro results are contrary to the previously presented in vivo study of Takeuchi et al. [94]. Further studies are needed to completely elucidate the influence of the xCT system in GBM progression and treatment.

Cancer cells have an increased metabolic activity and an increased production of ROS that often requires the overexpression of antioxidant pathways. Therefore, the xCT system is important in cancers and presents as a valuable target in anti-cancer therapy, including GBM [101]. Overexpression of the xCT system is involved in GBM cell growth and survival to therapy by increasing the mitochondrial biogenesis and ATP generation, by reducing ROS formation [102]. Via xCT involvement, ferroptosis takes an important part in regulating the GBM response to radiation, TMZ and immuno-therapy. All of these therapies suppress xCT and potentate tumoral cell death, guiding towards a possible better outcome for GBM patients [103,104,105].

In GBM cells, Gao et al. found that ibuprofen could inhibit the cells viability via increasing lipid peroxidation and ferroptosis. The treatment also downregulated xCT and GPX4 expression [19]. Using another drug repurposing approach, Sleire et al. found that sulfasalazine inhibited xCT activity in glioblastoma human xenografts in mice. This potentiated the effects of radiation therapy by increasing ROS and depleting GSH, improving the overall survival of the animals [106]. Similar results were found by other authors. Associating sulfasalazine treatment with valproic acid increased intracellular ROS and induced cell death, and sulfasalazine also increased TMZ cytotoxicity in vitro [107,108]. TMZ induces xCT expression in GBM treated cells, probably as a compensatory mechanism for the increase in ROS. Erastin xCT inhibition, together with TMZ proved to be more efficient than erastin or sulfasalazine alone [109]. In human subjects, Takeuchi et al. showed that the usage of sulfasalazine is associated with a high degree of hematologic toxic effects, while not providing a significant increase in survival [110]. Although sulfasalazine might not be efficient in GBM patients, xCT inhibition appears as to be potential target for future therapies associated with current radio- and chemo-therapeutic protocols, and could thus improve patient outcome [111].

Similar results have also been studied in GBM cancer stem cells. xCT overexpression might contribute to tumour progression and survival, while its inhibition decreases cancer stem cells invasion, survival and self-renewal abilities [112,113,114].

### 5.3. Lipid Peroxidation

PE-PUFAs are vital for ferroptosis, as they are the main substrate for lipid peroxidation [31]. ACSL4 is an acyl-CoA synthetase involved in the process of PE-PUFA synthesis and therefore is a key component in ferroptosis [115]. One of the main characteristics of GBM is represented by the tumour necrosis [116]. In this process, one mechanism is represented by neutrophils-induced ferroptosis. ACSL4 inhibition is associated with diminished necrosis areas and a less aggressive tumour behaviour. Thus, ferroptosis has a pro-tumorigenic role in early tumour progression by participating in the necrosis process, further enhancing the process by neutrophils recruitment that further increase ferroptosis and necrosis [117]. Suppressing the ACSL4 pathway reduces tumour progression and, furthermore, it increases the tumour sensibility to TMZ treatment [118].

Lipoxygenase (LOX) activity catalyse the oxygenation of PE-PUFAs into lipid peroxides that further activate signalling pathways or are used as substrates for further lipid mediators [119]. Additionally, iron plays the role of an enzyme effector for LOXs activity, especially 15-LOX isoforms, which plays a significant part in generating lipid peroxides [120]. In cancer therapy 15-LOX-1 stimulation seems to have a beneficial effect in reducing cancer growth and progression in vitro [121,122,123,124]. In GBM, 15-LOX upregulation by silencing IL-13Rα2 reduces tumour growth and promotes apoptosis [125].

MicroRNA (miRNA) 17-92-cluster up-regulation is involved in the pathogenesis of GBM [126]. A member of this cluster, miR-18a, is highly expressed in human GBM cell lines and regulates tumour cells progression, migration and invasion abilities [127]. MiR-18a also down-regulates ALOXE3 activity, reducing ferroptosis and thus providing a survival advantage in GBM. Inhibition of the miR-18a/ALOXE3 axis could provide a beneficial therapeutic approach [128].

The previously discussed molecules and pathways have a pivotal role in ferroptosis; however, its main regulatory enzyme is represented by GPX4. GPX4 is directly involved in transforming lipid peroxides into lipid alcohols by using GSH as a substrate. In its absence or reduced activity, lipid peroxides accumulate and induce lipid membrane damage that leads to cell death [129]. In cancer treatment, GPX4 modulation via influencing ferroptosis plays a crucial role in cancer cell death [130,131]. Absence or reduced expression of this molecule is also increasing 15-LOX activity, that not only increases the ROS availability, but also increases the tumour angiogenesis by a VEGF independent pathway [132,133].

Dihydroartemisinin (DHA) was studied as a potential GBM inhibitor by inducing apoptosis, autophagy and by suppressing the invasion ability of GBM cells [134,135]. Recently, the DHA inhibitory effect in GBM has been associated with ferroptosis. DHA significantly reduces GPX4, while maintaining ACSL4 and xCT system activity, increases ferroptosis and causes cancer cell death [54]. Treatment with a curcumin analogue reduced GPX4 activity, reducing GBM cells growth and TMZ resistance in vitro and improving survival in vivo in experimental rodents [136].

## 6. Conclusions

**Figure 2:**
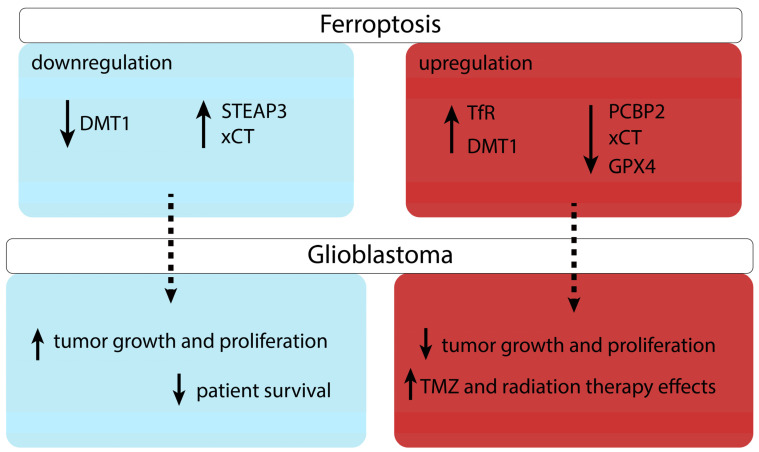
Ferroptosis modulation in glioblastoma. Abbreviations: DMT1, divalent metal transporter 1; GPX4, glutathione peroxidase 4; PCBP2, poly(rC)-binding protein 2; STEAP3, six-transmembrane epithelial antigen of the prostate 3; TFR, transferrin receptor; TMZ, temozolomide; xCT, cystine/glutamate antiporter.

GBM remains one of the deadliest tumours, with a poor prognostic even considering current therapeutic efforts. By being a non-caspase dependent form of cell death, ferroptosis presents as a promising process that could be involved in cancer treatment. By inducing it in GBM, cancer cells growth and differentiation is inhibited and also, the response to radiation and to TMZ treatment is marginally improved. Inhibiting the xCT system, reducing cysteine levels and thus GSH levels, as well as reducing GPX4 activity and increasing iron availability and ROS formation, each stimulate lipid peroxidation and thus, promote ferroptosis that in turn limits the cells’ ability to survive and to develop mechanisms of resistance to treatments (Figure 2). Future studies should test whether ferroptosis inducers have a real clinical impact on cancer patients, as adjuvant therapy to current standard therapies.

## Data Availability

Not applicable.
